# What Circuits, Masks and Filters Should Be Used in Home Non-Invasive Mechanical Ventilation

**DOI:** 10.3390/jcm12072692

**Published:** 2023-04-04

**Authors:** Manel Luján, Pablo Flórez, Xavier Pomares

**Affiliations:** 1 Servei de Pneumologia, Hospital Universitari Parc Taulí, 08208 Sabadell, Spain; pflorez@tauli.cat (P.F.); jpomares@tauli.cat (X.P.); 2 Centro de Investigacion Biomédica en Red (CIBERES), 28029 Madrid, Spain

**Keywords:** rebreathing, intentional leakage, active valve, interfaces

## Abstract

Most of the published reviews about non-invasive home ventilation mainly reflect the technical aspects of ventilators. There is much less information about the consumables most used at home. However, the choice of a good interface or tubing system can lead to physiological changes in the patient–ventilator interaction that the clinician should be aware of. These physiological changes may affect the performance of the ventilator itself, the reliability of monitoring and, of course, the comfort of the patient. The use of different circuits, masks or filters is therefore related to the concepts of rebreathing, compressible volume, instrumental dead space or leak estimation and tidal volume. Through certain bench experiments, it is possible to determine the implications that each of these elements may have in clinical practice.

## 1. Introduction

The huge spectrum of non-invasive home ventilation devices offers to the clinician a wide range of technical prescription possibilities for the management of chronic respiratory failure secondary to certain diseases (mainly COPD, obesity–hypoventilation syndrome, neuromuscular and restrictive diseases) [1,2,3]. These possibilities are not only restricted to the device, mode of ventilation or parameters, but also are extended to the so-called consumables that include a good number of different interfaces, filters, and circuits. One can think that the choice of these elements is not important, but it can be associated to important changes in the ventilator performance. The implications of a lack of knowledge of the features of the consumables can have a negative effect on the quality of ventilation. This review will outline the characteristics of these items, their implications on the physiology of ventilation and the potential complications that may arise from a poor choice of any of them.

## 2. Circuits and Expiratory Valves

The ventilator delivers the air generated inside the device to the patient through a circuit or tubing, which connects the interface in the proximal end with the ventilator at the distal end. As the choice of one or the other circuit model is closely linked to the system for preventing rebreathing of the patient’s exhaled gas, both concepts will be discussed together. Therefore, there are three main possible combinations of circuit and anti-rebreathing system: the dual limb, the single limb with active valve and the single limb circuit with intentional leak [4]. For each kind of circuit, the pathophysiological implications will be analysed based on the following issues: the effectiveness in preventing rebreathing, the impact on ventilator performance and the potential impact on monitoring or estimating main ventilatory parameters (mainly tidal volume and leakage).

### 2.1. Double Limb System

The dual limb circuit is made up of two individualised tubings, inspiratory and expiratory. Both are connected to the ventilator at one end and to a Y-piece at the other end, which in turn is connected to the interface. The distal (ventilator) ends are connected to inspiratory and expiratory non-return valves inside the device. This setup, with two unidirectional valves, ensures that the gas delivered to the patient during inspiration is not lost through the expiratory limb, and conversely, the patient’s exhaled air never flows through the inspiratory limb. Therefore, such a configuration does not require additional anti-rebreathing devices (active valves or intentional leak systems) and needs few corrections (see below) for a precise tidal volume or leakage estimations. However, is often considered too bulky for home environment.

#### 2.1.1. Implications for Monitoring

Tidal volume: Usually, tidal volume is displayed from the measures at both ends of the inspiratory and expiratory limbs. The direct measurement of inspiratory and expiratory flows (and volume) by the internal pneumotachographs needs to be corrected only by the compressible volume, that is the amount of gas (in mL) compressed in the circuit for every cm H_2_O of pressure generated by the ventilator during the inspiratory phase. This volume never reaches the patient and usually depends on the compliance (ΔV/ΔP) and length of the circuit and pressure difference inside and outside the tubing. Depending on these factors, the compressible volume can represent up to 20% of delivered gas [5]. The compressible volume implications are also different depending on whether a pressure mode or a volumetric mode is used. In pressure mode, there is an underestimation of the true tidal volume, as the ventilator interprets that the entire monitored volume is delivered to the patient, regardless of the volume compressed in the tubing. In volumetric mode, the programmed tidal volume is not fully delivered to the patient (up to 20% can remain as compressed volume). Modern ICU ventilators are usually fitted with algorithms that estimate and compensate delivered flow for compressible volume. A good example of a misleading compressible volume is observed in situations with large pressure differentials, such as mechanical in-exsufflation, in which pressure differences up to 80 cm H_2_O can lead to flow artifacts during these sudden pressure changes [6].

It should also be noted that, in case of leaks, the inspired volume may be different from the exhaled volume, and it should be known which of the two values is displayed on the screen (see below). Finally, for leakage estimation, in double limb configuration, the difference between inspired and expired volume (previously corrected by the compressible volume) corresponds to the non-intentional leakage.

#### 2.1.2. Implications for Ventilator Performance

It is particularly important to determine the estimated reference volume used by ventilators when prescribing volume-assured hybrid modes: the reference system on the inspiratory or expiratory limb can lead to dysfunctions, especially in case of leaks. If the reference volume is taken on the output pneumotachograph (inspiratory limb), the existence of leaks may overestimate the tidal volume, inducing the system to decrease the pressure support. This phenomenon does not occur if the reference tidal volume is taken on the expiratory limb, although the existence of leaks during the expiratory phase could also lead the system to underestimate the actual tidal volume and inappropriately increase the pressure support.

### 2.2. Single Limb with Active Valve

In the active valve circuit, a pneumatic device is usually connected between the proximal end of the limb and the interface, acting as a true expiratory valve. During the inspiratory phase of the cycle, the valve is pressurized above pressure inside the limb. This pressure increase occludes the valve during inspiration and prevents the leakage of delivered gas during inspiration. Conversely, during expiration, the pressure valve relief causes the pressure inside the tube to become higher than the pressure in the valve, opening the valve and allowing the exit of the exhaled gas [4].

**Figure 1:**
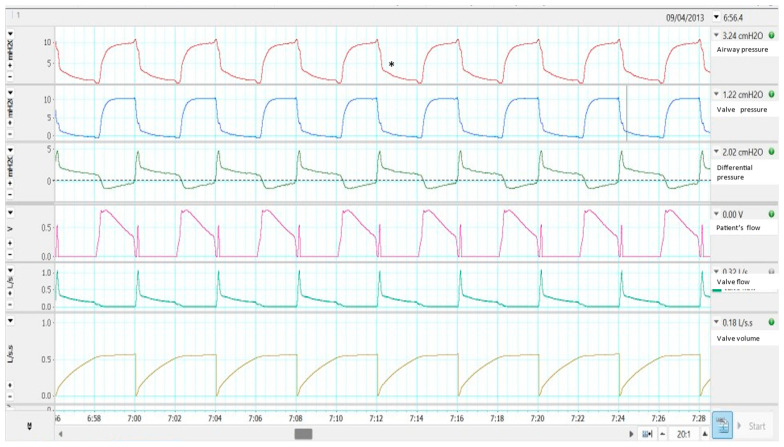
Performance of a single limb system with active valve in a bench test. See the text for more details. Asterisk displays the slower despressurization in the airway pressure than in the valve (channel 2).

Figure 1 reproduces an experiment explaining the pressure and flow physiological changes during the use of this kind of circuit in a bench environment. Briefly, for this experiment, an active simulator and an external monitoring system were used. The external monitoring system included two different pressure sensors that monitored pressure inside the limb (Channel 1, in red) and in the valve, through an interposed three-way stopcock (Channel 2, in blue). In channel 3 (green), the difference between both pressures (Channel 1–Channel 2) was calculated and continuously displayed: a value lower than zero (dotted line) during inspiration turned to a sudden peak at the beginning of expiration, which allows the opening of the valve. This peak seems to be produced by a slower pressure release in the pressure inside the tubing (Channel 1, asterisk) than in the valve (Channel 2). The fourth channel (purple) represents the monitored flow: it is interesting to see the sudden positive flow at the beginning of expiration, corresponding to the previously mentioned pressure gradient. It is also noteworthy that during expiration, the signal became completely flat because expiratory flow escaped through the pneumatic valve. Finally, Channels 5 and 6 represent the exhaled flow and volume, measured by a second pneumotachograph attached to the expiratory valve.

#### Effects on Monitoring

The expiratory valve coupled to the single limb system was used long ago as a reference for monitoring the patient’s exhaled volume by means of an attached Wright spirometer [7]. The necessary condition to ensure the reliability of such a determination was ventilation without PEEP, as the use of PEEP needs an expiratory flow from the ventilator that would provide wrong lectures (overestimation) in the exhaled tidal volume.

In the modern built-in software, this kind of circuit makes it very difficult to estimate the tidal volume in presence of leakage, as the reference for the leakage estimation comes from the expiratory part of the flow (see below), and this information is missing. Moreover, as demonstrated by Carlucci et al. [8], pressure support systems with assured volume are particularly unreliable with this setup. In this setting, the only technical solution available for measuring TV would be to place a pneumotachograph between the valve and the interface.

**Figure 2:**
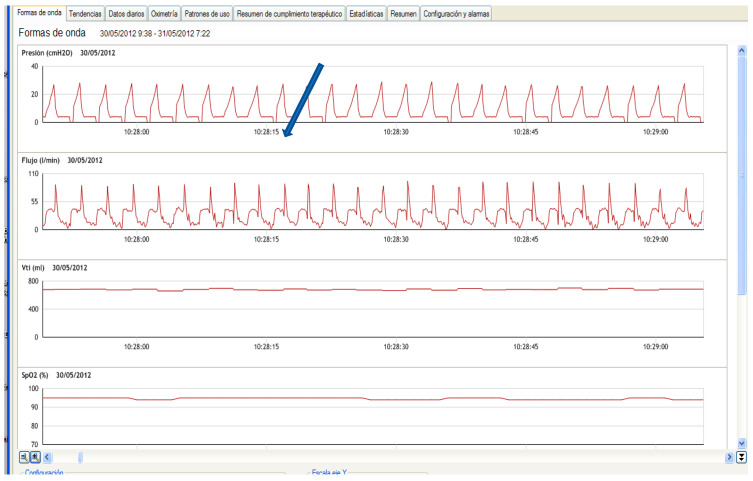
Example of a built-in software in volume mode and a single limb system with active valve. Channel 1: Pressure, Channel 2: Flow. The arrow is indicating the flow increase corresponding to the active valve opening. Channel 3 corresponds to the estimated tidal volume and channel 4 to the oxygen saturation.

Finally, as the exhaled tidal volume is not detected by the ventilator’s integrated pneumotachograph, there is a lack of information about what happens in the expiratory phase of the cycle. In addition, some built-in software displays the valve opening spike on the screen (in the flow/time waveform), which may lead to misinterpretation by the clinician. An example of this semiology is displayed in Figure 2.

### 2.3. Single Limb with Intentional Leakage

It is the most preferred configuration in home NIV, and due to its simplicity, its setup is easy for both patients and caregivers [9,10]. The intentional leakage can be built in the whole mask or as an independent piece between the mask and the tubing. Although it is the less bulky system, the gas flow dynamics are complex and deserve to be analysed in depth.

From a generic point of view, the expiratory port acts as a continuous and intentional leakage system, with a higher resistance than the imposed by the diameter of the limb. Therefore, during inspiration, the gas delivered by the ventilator is divided into two parts: the tidal volume received by the patient and the flow lost through intentional leakage [11].

**Figure 3:**
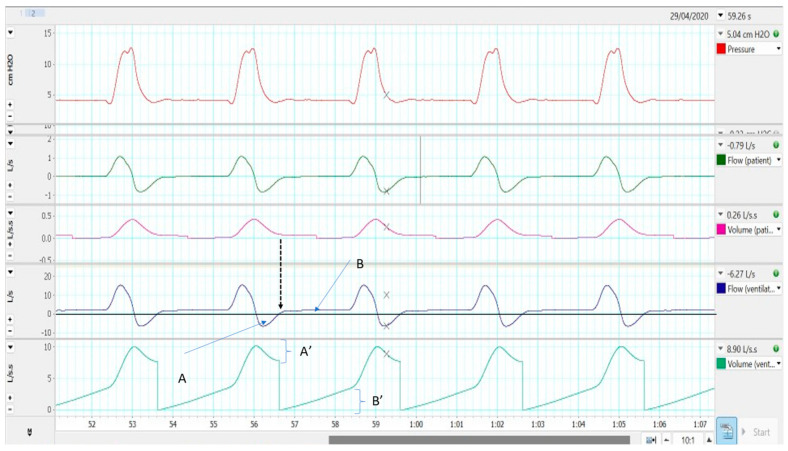
Gas flow dynamics in a bench model of single limb with intentional leakage. See the text for more details.

Conversely, during expiration, the gas exhaled by the patient is partly lost through intentional leakage, but at the same time, there is a variable amount of flow (because of the lower resistance imposed by the tube) that flows back through the tube and is detected by the pneumotachograph inside the ventilator. The performance of flow dynamics in single limb with intentional leakage is reflected in Figure 3. In this experimental set-up, two pneumotachographs were used, before and after the leakage: the one proximal to the simulator (Channels 2 and 3) reads the true flow/tidal volume entering the simulator. The distal one (Channels 4–5) displays “what the ventilator is reading”. Continuous arrow in Channel 4 corresponds to the backward flow, whereas dotted arrow points out the inversion from negative to positive flow. Channel 5 corresponds to the integration of Channel 4 (volume): backward volume can be easily calculated from the maximum volume to the transition point (A’ in the figure), in which there is a “restart” (sudden drop, asterisk) of the integral. Usually, this restart is programmed in the transition point from negative to positive flow (in the dotted arrow point). From this restart, all the positive volume until next breath start would correspond to the “washout flow” (patient is remaining in expiration; therefore, the only point at which this flow can exit is through the intentional leakage). This volume has been labelled as B’ in Figure 3.

#### 2.3.1. Effectiveness in Preventing Rebreathing

The fact that the inspired gas is not separated from the exhaled gas (as is the case with the double tube) or the absence of a real expiratory valve seems a priori to favour the rebreathing of a part of the patient’s exhaled gas.

**Figure 4:**
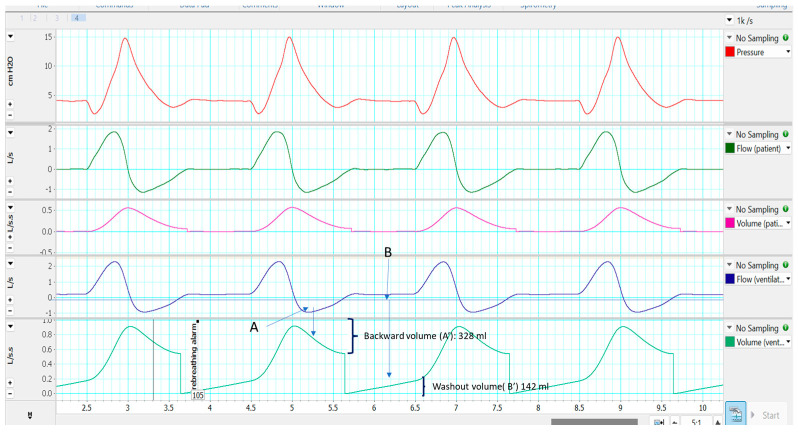
Example of rebreathing. See the text for more details.

From Figure 3, it can be deduced that the backward flow (continuous arrow) would contain a volume of CO_2_ exhaled from the patient. That volume must be washed out from the point at which the gas flow reverses and becomes positive (“washout flow”). From the integral of the two volumes (Channel 5), it can be easily determined whether there is an imbalance between the two volumes that favour rebreathing. If backward volume is clearly greater than washout volume, rebreathing might occur. In Figure 4, there is an example of rebreathing, comparing backward flow and washout flow, even with activation of the rebreathing alarm in the ventilator.

**Figure 5:**
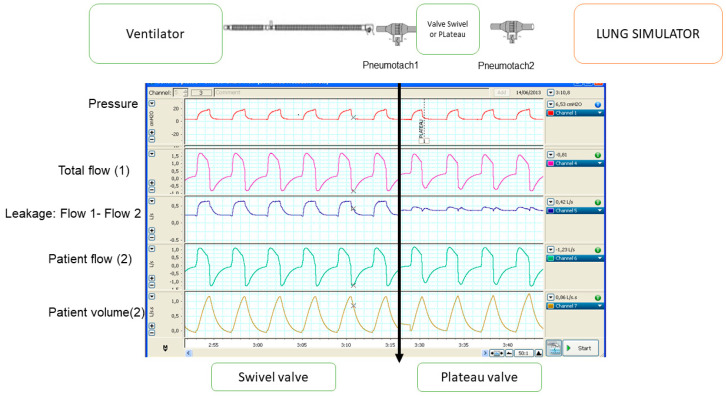
Comparison of intentional leakage between Swivel and Plateau valves. See the text for more details.

This relationship between the aforementioned areas may change depending on several parameters: the PEEP used in the system (the higher the PEEP, the higher the valve washout flow and the lower the backward flow), the patient’s expiratory time (the shorter the expiratory time, the lower the opportunity for washout), the patient’s tidal volume, and finally the valve flow at equal PEEP. Regarding this last point, it has been determined that in patients with ventilation under effort, which seems to be a condition that favours rebreathing due to the shorter expiratory time (tachypnoea) and the higher tidal volume, the so-called Plateau™ valve is less likely to rebreathe than the Swivel™ valve [12]. In a bench experiment, again with two different pneumotachographs before and after the intentional leakage (Figure 5), it seems clear that the washout (expiratory) flow at equal PEEP is considerably higher in the Plateau™ valve than in the Swivel™ valve. At same time, and due to the oscillatory movement of the membrane built inside the plateau valve, the leakage during inspiration is lower in the Plateau™ valve than in the Swivel™ valve. This special performance of the plateau valve could eventually lead to misestimation in patient’s tidal volume if this kind of expiratory port is used in a ventilator that does not recognize its performance.

**Figure 6:**
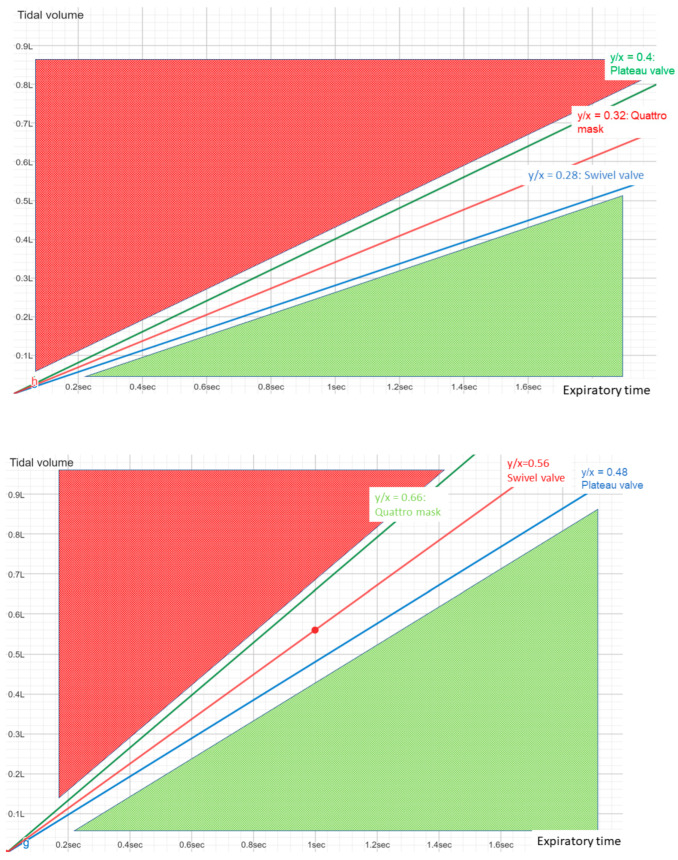
Estimated likelihood of rebreathing of three different types of intentional leakage. The slope for each mask corresponds to the amount of intentional leakage at PEEP = 4 cm H_2_O (upper panel). For any further increase in PEEP, an increase in the slope would be expected (lower panel, PEEP = 8). Red zone represents the high risk for rebreathing, whereas green zone represents the “safe” zone which increases with higher PEEP.

Examining the data summarised graphically (Figure 6), we could say that for any combination of tidal volume and expiratory time, knowing the flow through the valve at the PEEP used, it is possible to determine “safe” zones with a low probability of rebreathing and less safe zones with a higher probability. The PEEP level used only modifies the slope of the straight line that delimits the safe and unsafe zones.

There are few studies analysing rebreathing from a clinical point of view. Schettino et al. demonstrated that the likelihood of rebreathing is lower if the leak port is included in the mask [13] Finally, Szkulmowski et al. demonstrated that factors such as PEEP level, tachypnoea, and initial EtCO_2_ had an influence on rebreathing [14]. It has been recently proposed that a novel mask design, with separate limbs for inflow and outflow gases, significantly reduced CO_2_ rebreathing in different ventilation settings [15]. In the clinical practice, the clinician often feels comfortable using a low level of EPAP (around 4 cm H_2_O) for preventing rebreathing, but the complex interactions between patient’s inspiratory pattern, leakage bias flow and pulmonary mechanics leading to rebreathing should not be automatically discarded as a cause of NIV failure [16].

#### 2.3.2. Influence on Monitoring

The complexity of gas dynamics and the monitorization inside the ventilator make impossible the direct determination of tidal volume. Instead of direct measure, leakage and tidal volume must therefore be estimated from the data monitored at the ventilator’s internal pneumotachograph.

The correct estimation of tidal volume and thus of intentional and unintentional leakage in single tube systems must take several aspects into account [11]:

- Both parameters (tidal volume and leakage) are closely related, as the total flow is the sum of tidal volume plus the leakage. Therefore, if one is underestimated, the other will also be overestimated.
- The reference point commonly used to determine leakage (and indirectly tidal volume) is usually the transition between expiration and inspiration, because at that point, the patient’s flow is zero and therefore all circulating flow will correspond to leakage. In fact, it is analogous to a single equation with two unknowns, in which the total amount of flow is known for the entire cycle, but their components (patient’s flow and leaks) are not known. Only in two points of the cycle (transitions from inspiration to expiration and vice versa) one of the values (leakage) is known since the patient’s flow is zero. The choice of the expiratory–inspiratory transition as a reference is related to the flatter slope in the flow waveform at this point. Then, small deviations in the curve would not represent a big error in the estimations.
- If the leak flow and the pressure (EPAP/PEEP) are known, the resistance of the system during the expiratory phase can be estimated by means of the Poiseuille’s law for fluids. Usually, the leakage during inspiration is calculated by applying the resistance value to the inspiratory phase. In other words, a linear or near-linear relationship is assumed. However, there are some situations in clinical practice that do not fulfil this linearity (asymmetric leaks). For example, a poorly attached interface can produce leakage in the system only during inspiratory phase. In this case, the leakage (and the tidal volume) can be wrongly estimated [17].
- Based mainly on this single datum (resistance during expiratory phase), an attempt is made to determine what happens for the whole respiratory cycle. Therefore, if this point is taken as a reference (at the end of the cycle), the analysis must be applied to the next cycle, not to the same cycle that generated this datum. This can be misleading in very heterogeneous cycles.
- The estimation of leakage (and therefore tidal volume) must consider that the actual data (pressure and flow) are obtained inside the ventilator, and that there is a pressure loss through the system dependent on the outlet flow and the properties of the tubing (resistance and compliance, by Poiseuille’s law). Some manufacturers have incorporated tests for the correction of the pressure values (pre-use tests) for greater reliability in the estimation of the tidal volume [18]. Another option would be the placement of a proximal pressure sensor, such as in some acute care ventilators.

#### 2.3.3. Influences on Ventilator Performance

The drawbacks of this misestimation can go beyond wrong information to the clinician. It should be highlighted that in some new modes of NIV (the dual control or volume-targeted pressure support modes), the ventilators make their own decisions based on target TV estimation. If asymmetric leaks appear as demonstrated in a bench study, the ventilator can modify its pressure support level in a different way than expected. In this setting, it seems reasonable to recommend setting a safety lower level of pressure support in the ventilator [19].

## 3. Interfaces

This is one of the key elements in long-term non-invasive home ventilation. A wrong choice of interface can lead to failure of ventilation due to lack of patient comfort. There are enough commercial alternatives for most patients’ facial anatomical variants. Generally, home NIV interfaces can be divided according to the anatomical structure to which they fit. The most widely accepted classification is as follows [20]:

- Nasal masks: cover the nasal structure, with support on the nasal bridge and upper jaw area, especially.
- Nasal pillows: cover only the nasal orifices.
- Oronasal masks, whey can be classified into two subtypes: the conventional oronasal mask, with support on the nasal bridge and lower jaw, and the subnasal (“hybrid”) mask, with support on the lower jaw but respecting the nasal bridge by means of support on the cartilaginous area of the nose.
- Full-face masks: nose and mouth coverage and forehead support.
- Oral-only interfaces: mouthpiece and lip-seal masks.

In addition, most of them may or may not incorporate intentional leakage at the interface (vented or non-vented systems). It should be considered that if the intentional leakage is used as an external piece connector between the interface and the limb, the instrumental dead space will be greater [13].

Several variables can play a role in the choice of one or other model, related to the patient, the underlying disease to be treated with NIV, and even the personal preferences of the professional who indicates ventilation.

In terms of efficacy, a recent systematic review in patients with COPD and obese hypoventilation syndrome showed no difference in efficacy and tolerability between the two main mask models, nasal and oronasal [21]. However, prescriber preferences were mainly reported for the use of oronasal masks. Despite this preference, oronasal masks may cause posterior displacement of the lower jaw and tongue, increasing the occurrence of upper airway events, with higher expiratory pressure requirements for control [22]. This effect has also been demonstrated by MRI in patients with obstructive sleep apnoea [23]

In restrictive patients, including neuromuscular individuals, a recent international survey [10] showed that the preferred interface for nocturnal ventilation was the oronasal mask, while for daytime ventilation, the mouthpiece was the most used. Nevertheless, it is an interface that requires some skills to solve both the technical problems of adaptation and the side effects that patients may experience [24]. The choice of interface in neuromuscular patients is also influenced by factors related to the course of the disease. For example, if there is bulbar involvement, the patient may not be able to close their mouth properly, making it difficult to adapt to a nasal mask. If there is an increased need for ventilation hours, it is important to establish an interface rotation programme to avoid skin breakdown. Finally, if there are concomitant upper airway obstructive phenomena (common in Steinert’s myotonic dystrophy), the use of an oronasal mask may contribute to the decline in their condition through an increase in the number of upper airway events, by the previously described mechanism.

In COPD patients, the use of pressures high enough to normalise or at least decrease the basal PaCO_2_ by at least 20% (high intensity ventilation) has been established as the treatment of choice [2]. This phenomenon frequently leads to the use of inspiratory pressures higher than 20 cm H_2_O, with an increase in unintentional leakage, so the use of an oronasal mask seems to favour the management of this type of patient.

Taking up the original idea of customised masks [25], the use of masks made by 3D printing using a scan of the patient’s face has been proposed. In a pilot study, the use of these masks reduced the pressure on the mask support points at equal ventilator pressure [26]. Finally, Shikama et al. developed a 3D-printed adapter for patient fitting to a commercial mask. Volunteers who used this adaptor experienced lower leakage rates and greater comfort when using the NIV [27].

## 4. Filters and Other Items

Home ventilators are usually equipped with internal filters that protect the device from environmental particles. These filters should be changed at regular intervals. Viral–bacterial filters at the outlet of the ventilator are not usually used, as each ventilator is intended for the exclusive use of each patient. However, two recent events may have changed this practice. The first was the COVID-19 pandemic, during which many home ventilators were used in the acute care setting. To prevent the dispersion of viral particles into the atmosphere, the use of viral–bacterial filters was recommended. The addition of these filters could change the trigger sensitivity and pressurisation capabilities of the ventilator, as demonstrated on a bench test [28]. The second event was the notification by a manufacturer of the existence of polyurethane foam inside the device. This material could progressively become damaged with prolonged use and could eventually be inhaled by the patient, with adverse effects on their health. Therefore, the interposition of viral–bacterial filters in the inspiratory limb was recommended. However, a bench study showed that the addition of such filters had adverse effects on ventilator performance, with an increase in the product pressure time required to activate the trigger (PTPtrig), lower pressurisation and lower tidal volume [29]

Finally, should oxygen need to be added, there are two main technical possibilities for enriching inspired flow. The classical way is the interposition of a T-piece in the circuit, usually near the ventilator. However, it should be considered that, in this system, if the single limb with intentional leakage is used, the fraction of inspired oxygen at the mask is lower than the measured in the ventilator [30] and should be increased to compensate this loss, especially in presence of hypoxia [31]. However, the introduction of an external gas into the system may be related to the development of trigger asynchronies in certain models [32]. Nowadays, many home ventilators have separate oxygen inlets, which can tolerate flows of up to 30 litres per minute, sufficient for home use and even for ventilation in the acute care setting [33] and avoiding the same time some of the previously described drawbacks.

## 5. Conclusions

Good clinical practice in non-invasive ventilation extends to appropriate use of consumables (limb systems, interfaces and filters). The choice of appropriate items should consider the advantages and disadvantages of each one (summarized in Table 1). In addition to choosing an appropriate interface, the clinician should keep in mind the possibility of rebreathing in certain special circumstances (single limb with intentional leakage, patients with tachypnoea, high tidal volume, increased instrumental dead space or low PEEP values). Finally, the effects of the use of certain circuits on the reliability of monitoring should be taken into account, as they may lead to interpretation errors.

## Figures and Tables

**Table 1:** Summary of advantages and disadvantages of each type of consumable.

Item	Types	Advantages	Disadvantages
Limb systems	Double limb system	Accuracy in monitoring. only compressible volume needs correction	Bulky system
	Single limb with active valve	PEEP not strictly necessary	Flow expiratory waveform not displayed. Semiology may be confusing.
	Single limb with intentional leakage	Simple system	Rebreathing under certain conditions should be considered. Leakage and tidal volume estimation are challenging.
Masks	Nasal	Less instrumental dead space	Oral leakage.
	Oronasal	No oral leakage	Can worsen upper airway obstructions.
Filters		Protective effect	Interferences in trigger function and pressurization.

## Data Availability

Not applicable.
